# Mapping from SIBDQ to EQ-5D-5L for patients with inflammatory bowel disease

**DOI:** 10.1007/s10198-023-01603-9

**Published:** 2023-06-27

**Authors:** Isa Maria Steiner, Bernd Bokemeyer, Tom Stargardt

**Affiliations:** 1 Hamburg Center for Health Economics, University of Hamburg, Esplanade 36, 20354 Hamburg, Germany; 2 Interdisziplinäres Crohn Colitis Centrum Minden, Märchenweg 17, 32429 Minden, Germany

**Keywords:** Short Inflammatory Bowel Disease Questionnaire, SIBDQ, EQ-5D, EQ-5D-5L, Mapping, Preference-based measure, I1, C3

## Abstract

**Objective:**

Clinical studies commonly use disease-specific measures to assess patients’ health-related quality of life. However, economic evaluation often requires preference-based utility index scores to calculate cost per quality-adjusted life-year (QALY). When utility index scores are not directly available, mappings are useful. To our knowledge, no mapping exists for the Short Inflammatory Bowel Disease Questionnaire (SIBDQ). Our aim was to develop a mapping from SIBDQ to the EQ-5D-5L index score with German weights for inflammatory bowel disease (IBD) patients.

**Methods:**

We used 3856 observations of 1055 IBD patients who participated in a randomised controlled trial in Germany on the effect of introducing regular appointments with an IBD nurse specialist in addition to standard care with biologics. We considered five data availability scenarios. For each scenario, we estimated different regression and machine learning models: linear mixed-effects regression, mixed-effects Tobit regression, an adjusted limited dependent variable mixture model and a mixed-effects regression forest. We selected the final models with tenfold cross-validation based on a model subset and validated these with observations in a validation subset.

**Results:**

For the first four data availability scenarios, we selected mixed-effects Tobit regressions as final models. For the fifth scenario, mixed-effects regression forest performed best. Our findings suggest that the demographic variables age and gender do not improve the mapping, while including SIBDQ subscales, IBD disease type, BMI and smoking status leads to better predictions.

**Conclusion:**

We developed an algorithm mapping SIBDQ values to EQ-5D-5L index scores for different sets of covariates in IBD patients. It is implemented in the following web application: https://www.bwl.uni-hamburg.de/hcm/forschung/mapping.html.

**Supplementary Information:**

The online version contains supplementary material available at 10.1007/s10198-023-01603-9.

## Introduction

The prevalence of inflammatory bowel disease (IBD), including Crohn’s disease (CD), ulcerative colitis (UC) and indeterminate colitis (IC), has been rising in high-income countries since the mid-twentieth century [1]. In Europe, approximately 0.4% of the population has an IBD [2]. First diagnosis of the disease usually occurs in early adulthood [3], and there is currently no cure for it. The goal of treatment is to maintain health-related quality of life and avoid disability, which implies inducing remission in the short term and maintaining it in the long term [4, 5].

The number of treatment options for IBD patients has increased substantially in recent years. In particular, advanced medical therapies, such as biologics, have facilitated the management of patients with complicated disease courses [6]. Many of the new treatment options are, however, very costly, raising questions about their affordability for health care systems. Health economic evaluations are a useful tool for weighing the health outcomes of different treatment options against their associated costs. The most frequently used health measure for this purpose is the quality-adjusted life-year (QALY), which seeks to reflect both the length and quality of a patient’s life [7].

Calculating QALYs requires preference-based measures, such as the EQ-5D-5L index score, as input parameters [8]. Because such index scores are generic utility measures, they can be compared across different disease types. This generalisability often comes at the price of a lower sensitivity than disease-specific quality of life measures [9–11], which is why the latter are often preferred in clinical trials. However, the results of clinical studies that report only disease-specific measures are of limited use for cost-utility analysis. In such situations, mapping from the disease-specific quality of life measure to a utility index score can help overcome this problem.

In the context of IBD, the Inflammatory Bowel Disease Questionnaire (IBDQ) and its short version, the Short Inflammatory Bowel Disease Questionnaire (SIBDQ), are the most widely used disease-specific quality of life instruments for adult patients [12]. While a mapping already exists for the IBDQ [13], to our knowledge, no study has been published so far that maps the SIBDQ to a utility index score. Our aim was to close this gap by developing an algorithm for mapping values from the SIBDQ to EQ-5D-5L index scores. To do so, we applied regression and machine learning models for different sets of covariates, thereby taking differences in data availability into account. Moreover, we developed and provide an online tool that allows for a user-friendly application of the final mapping algorithm.

## Methods

This mapping study complies with the Mapping onto Preference-Based Measures Reporting Standards (MAPS) checklist [14]. Details are provided in Online Appendix 2.

### Data

Our study is based on data from the German multicentre randomised controlled trial CED_Bio-Assist_. The trial investigated the impact of introducing regular appointments with an IBD nurse specialist on patients’ quality of life and other outcome parameters among IBD patients in addition to standard care with biologics. It was implemented under real-life conditions.

Ethical approval was obtained. The registration ID in the German register for clinical trials is DRKS00020265. To be eligible for inclusion, patients had to be diagnosed with an IBD, at least 18 years old and either receiving an ongoing biologic therapy or scheduled to start a biologic therapy after inclusion. Inclusion took place between 6 January 2020 and 18 January 2021. Patient-reported outcomes and disease-related information were collected via patient and physician questionnaires at baseline, as well as 6, 12 and 18 months after baseline. This time interval corresponded to the usual distance between appointments in standard care of IBD patients in Germany. Remote and on-site monitoring took place to optimise data quality.

The data set contained 3999 observations of 1066 patients. After we deleted observations with missing values for any of the variables considered in the study, the data set contained 3856 observations of 1055 patients. The data was then randomly split into a model subset (80%) with 3066 observations of 844 patients and a validation subset (20%) with 790 observations of 211 patients.

### SIBDQ

The SIBDQ is the short version of the 32-item IBDQ [15, 16]. It consists of 10 items belonging to four different domains: systemic, social, bowel and emotional. Each item is answered on a Likert-type response scale with 1 and 7 representing the lowest and highest quality of life, respectively. We defined the general score as the mean of the scores of all 10 items. Moreover, we calculated subscales for each of the four domains as the mean of the scores of the respective items. The SIBDQ is available in multiple languages, including English, German, Spanish, Portuguese, French and Mandarin. Validation studies showed good reliability, validity and responsiveness [12, 17–19].

### EQ-5D-5L

The EQ-5D-5L is a generic instrument that measures health-related quality of life. Its major components are five Likert-type response scales between 1 and 5 in each dimension. There are three functional dimensions (mobility, self-care and usual activities) and two somatic symptom dimensions (pain/discomfort and anxiety/depression) [20]. The responses to the five scales can be translated into a single utility index score. This index score is based on a country-specific value set, which is usually obtained with a discrete choice experiment, time trade-off or standard gamble approach. The German value set, which is the basis for our analysis, ranges from − 0.661 to 1 [21]. In general, the distribution of the index score is skewed to the left with many observations being close or equal to 1 [22]. While there are still few studies assessing the responsiveness of the EQ-5D-5L, its reliability and validity have been assessed in multiple publications, suggesting that it can be applied in a wide range of populations and settings [23].

### Covariates considered for mapping

The main goal in mapping studies is to estimate the relationship between two quality of life measures. However, there are other factors that might affect health-related quality of life and therefore improve predictions and generalisability when included in the estimation process [24]. In the case of IBD, female gender and age were found to be negatively associated with EQ-5D-5L index scores in a representative population sample in Germany [25]. Moreover, the body mass index (BMI) is considered to affect health-related quality of life, especially in the presence of obesity [26]. Smoking status has different implications across IBD subgroups: while continued smoking is a risk factor for adverse outcomes in CD patients, it may be protective against adverse outcomes in UC patients [27]. Finally, previous studies indicate that quality of life may improve over time after an IBD has been diagnosed [9].

To allow for mapping depending on data availability, we assumed five different scenarios: (1) Only the general SIBDQ score is mapped to EQ-5D-5L; (2) The general SIBDQ score and the demographic variables age and gender are available for mapping; (3) Additionally, the patient’s BMI, smoking status and type of IBD (i.e., CD vs. UC or IC) are known; (4) In addition to the variables included in scenario 3, the number of years since the patient’s first IBD diagnosis is known; (5) In addition to the variables included in scenario 4, the SIBDQ subscales are available for mapping.

### Estimated models

Since our data contained repeated measurements, we estimated the models considered for mapping with random intercepts by individual. The following four approaches were used to map the SIBDQ to the EQ-5D-5L: First, we estimated linear mixed-effects regressions (LMER) because linear regression is the most common approach in studies mapping disease-specific measures to EQ-5D index scores [28]. Second, we ran a mixed-effects Tobit regression, which is another common approach for estimating utility index scores [28, 29]. Tobit regression is an extension of classical regression approaches that was designed for dealing with limited dependent variables [30]. Third, we ran an adjusted limited dependent variable mixture model (ALDVMM), which was specifically developed to estimate regressions with the EQ-5D index as dependent variable [31]. The ALDVMM was estimated with one component. The lower and upper limits were set to − 0.661 and 0.974, as they represent the lowest and second highest value in the German value set respectively. In contrast to the other models, we did not estimate random intercepts for ALDVMM, as this option is currently neither available in the R nor in the Stata package. We did, however, estimate cluster-robust standard errors, as suggested by Oliveira Gonçalves et al. [32]. Finally, we estimated our mapping algorithm using a mixed-effects regression forest (MERF). MERF consist of a random effect and a fixed part of the model, where the latter is estimated with a regression forest [33, 34]. Regression forest is an approach from machine learning that does not require an assumption about the distribution of the dependent variable or the functional form of the relationship between outcome and explanatory variables. It consists of many regression trees that split random samples of the data into different segments and make local predictions of the outcome variable. The final prediction for a given set of predictors is then obtained by averaging the predictions across multiple regression trees [35]. Online Appendix 2 contains a more detailed description of the estimated models.

Because it is not easy to decide whether and in which form specific variables should be included in regression models, e.g., whether quadratic, cubic or interaction terms are sensible, we specified LMER, Tobit and ALDVMM for each scenario in two different ways: the first (LMER), third (Tobit) and fifth (ALDVMM) method consisted of parametrising the model for each data availability scenario without polynomials and interaction terms. As a second (LMER′), fourth (Tobit′) and sixth (ALDVMM′) method, we applied the ‘lasso’ technique developed by Tibshirani et al. [36] to select variables that included third order polynomials of all continuous variables, as well as interaction terms of all variables. Thus, the models OLS′, Tobit′ and ALDVMM′ were specified with the variables selected by the lasso. We did not use lasso in addition to the last method (MERF) because the latter already conducts variable selection by definition. As a result, we estimated seven models (LMER, LMER′, Tobit, Tobit′, ALDVMM, ALDVMM′ and MERF) for each of the five sets of covariates as described above (see Table 1).

**Table 1:** Description of the estimated models

Covariates	Method 1 LMER	Method 2 LMER′	Method 3 Tobit	Method 4 Tobit′	Method 5 ALDVMM	Method 6 ALDVMM′	Method 7 MERF
1 General SIBDQ	Model 1.1	Model 2.1	Model 3.1	Model 4.1	Model 5.1	Model 6.1	Model 7.1
2 General SIBDQ, age, gender	Model 1.2	Model 2.2	Model 3.2	Model 4.2	Model 5.2	Model 6.2	Model 7.2
3 General SIBDQ, age, gender, BMI, smoking status, IBD type	Model 1.3	Model 2.3	Model 3.3	Model 4.3	Model 5.3	Model 6.3	Model 7.3
4 General SIBDQ, age, gender, BMI, smoking status, IBD type, years since first diagnosis	Model 1.4	Model 2.4	Model 3.4	Model 4.4	Model 5.4	Model 6.4	Model 7.4
5 General SIBDQ, age, gender, BMI, smoking status, IBD type, years since first diagnosis, SIBDQ subscales	Model 1.5	Model 2.5	Model 3.5	Model 4.5	Model 5.5	Model 6.5	Model 7.5

*ALDVMM* adjusted limited dependent variable mixture model*, BMI* body mass index, *IBD* inflammatory bowel disease, *LMER* linear mixed-effects regression, *MERF* mixed-effects regression forest, *SIBDQ* Short Inflammatory Bowel Disease Questionnaire

### Model selection and validation

For model selection and estimation, only the model subset was used. We selected one final model for each covariate scenario based on tenfold cross-validation, which has become increasingly popular for mapping studies in recent years (e.g., [37–41]). We thus randomly split the sample into 10 folds, i.e., equally sized parts of the data set. Subsequently, we estimated each of the models described in Table 1 based on the combined data of nine folds. We calculated performance measures based on the tenth fold, called the test set. Because there is no single measure that is clearly preferred in the literature to evaluate the performance of a model, we calculated multiple measures. More specifically, we chose the mean squared error (MSE), the mean absolute error (MAE) and the coefficient of determination R^2^. We also considered the range of predicted values to compare different models. The process was iterated until each of the 10 folds had served as a test set once. We then compared performance measures and selected the best performing model for each scenario, which subsequently was estimated using all observations in the model subset. A model was considered best if it outperformed the other models on at least two of the three performance measures. For a more detailed description of the estimation procedure of predicted utilities and a definition of the performance measures, refer to Online Appendix 2.

As a robustness check, we ran the best-performing models without some of the variables added at earlier stages (e.g., a scenario (5) model was run excluding age and gender). In the last step, we validated the predictions of these models using the validation subset.

All statistical analyses were performed in R version 4.2.3. The code can be found in Online Appendix 1. Moreover, we programmed a web application using the R package Shiny [42], for which more information is provided in Online Appendix 2.

## Results

### Description of the sample and conceptual overlap

Table 2 summarises the characteristics of the 1055 patients in the data set at baseline. Of these patients, 592 were diagnosed with CD and 448 with UC. The remaining 15 patients were diagnosed with IC, i.e., they may have presented with symptoms of both CD and UC. The mean age was 40.97 years and 52.42% of all IBD patients were female, which is comparable to other studies in Germany [43, 44]. On average, patients’ first IBD diagnosis dated back 12.15 years. The mean BMI of 25.63 was similar to the BMI of the total population in Germany [45]. Smoking was more common in patients with CD than in patients with UC, a finding that has also been found in other studies [46].

**Table 2:** Patient characteristics at baseline

	CD (n = 592)	UC (n = 448)	IBD (n = 1055)
	Ø (sd)/share	Ø (sd)/share	Ø (sd)/share
Age in years	40.73 (13.59)	41.32 (14.00)	40.97 (13.90)
Gender			
Female	57.77%	44.87%	52.42%
Male	42.23%	55.13%	47.58%
Currently smoking	24.16%	8.05%	17.17%
BMI	25.30 (5.33)	26.08 (5.42)	25.63 (5.36)
Years since initial diagnosis	13.48 (10.78)	10.48 (9.15)	12.15 (10.21)
EQ-5D-5L index score	0.84 (0.20)	0.88 (0.16)	0.86 (0.19)
SIBDQ			
General score	4.97 (1.18)	5.08 (1.24)	5.02 (1.21)
Bowel subscale	5.19 (1.26)	5.18 (1.39)	5.18 (1.32)
Emotional subscale	4.70 (1.42)	4.89 (1.37)	4.78 (1.40)
Social subscale	5.54 (1.58)	5.59 (1.65)	5.55 (1.61)
Systemic subscale	4.48 (1.43)	4.72 (1.45)	4.58 (1.45)

*BMI* body mass index, *CD* Crohn’s disease, *IBD* inflammatory bowel disease, *SIBDQ* Short Inflammatory Bowel Disease Questionnaire, *sd* standard deviation, *UC* ulcerative colitis

**Fig. 1:**
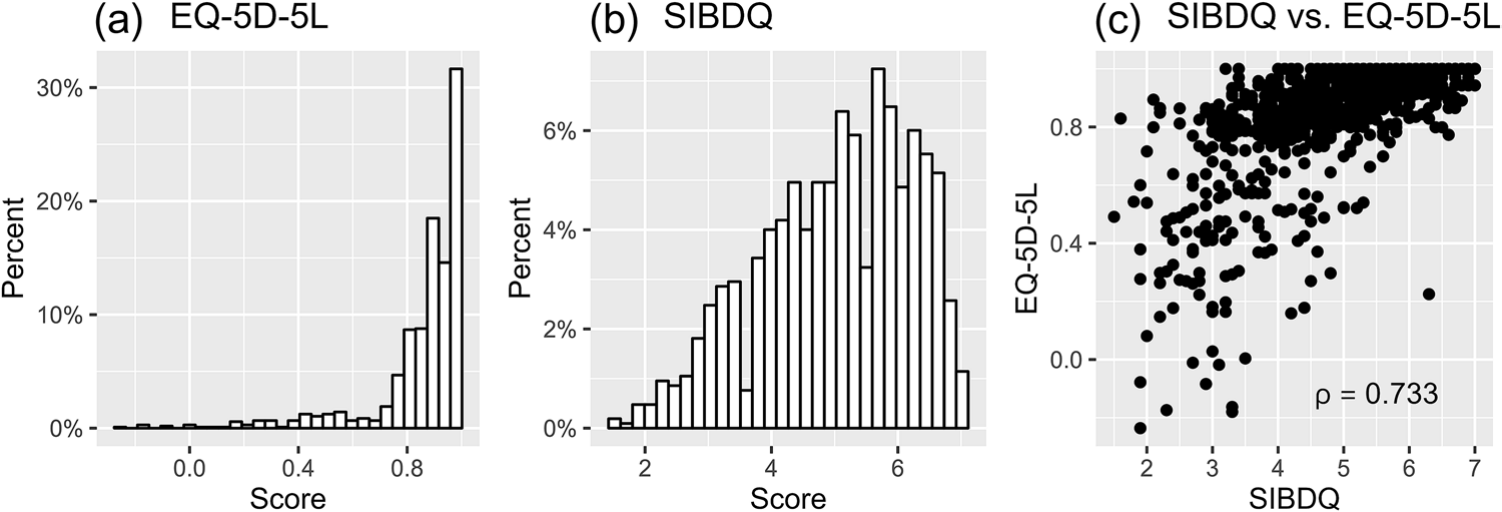
**a** Distribution of the EQ-5D-5L index score at baseline,** b** Distribution of the general SIBDQ score at baseline, **c** Scatterplot of the general SIBDQ score against the EQ-5D-5L index score at baseline; *SIBDQ* Short Inflammatory Bowel Disease Questionnaire, *ρ* Spearman correlation coefficient

The EQ-5D-5L index had a mean of 0.86 and a standard deviation of 0.19. The values ranged from − 0.236 to 1. Thus, no patient reached the lowest score possible. There was a strong ceiling effect, with 24.59% of all patients having an EQ-5D-5L index score of 1. The distribution had a skewness of − 2.58 and a kurtosis of 10.75, indicating that it was highly left skewed with few observations in the lower part. This is shown in Fig. 1a. Overall, the distribution was similar to that in other studies reporting EQ-5D-5L values for IBD patients [47–50].

The general SIBDQ score had a mean value of 5.02 and a standard deviation of 1.21, which is comparable to the values in other studies reporting this measure [50–53]. With the score ranging from 1.5 to 7, the lowest possible value was not reached. Approximately 1% of all patients had a general SIBDQ score of 7. Thus, the ceiling effect was smaller than that in the EQ-5D-5L. The distribution had a skewness of − 0.49, meaning that it was only slightly skewed to the left. This can be seen in Fig. 1b. Figure 1c shows a scatterplot of the general SIBDQ score against the EQ-5D-5L index score at baseline. The Spearman correlation coefficient of 0.73 was significant (p < 0.001), indicating a strong positive relationship between the two measures.

### Mapping algorithms

Table 3 shows the performance measures of the different models after tenfold cross-validation. The average predicted minimum (maximum) of LMER and LMER′ was above 0.55 (1.02). Tobit, Tobit′, ALDVMM, ALDVMM′ and MERF had a similar predicted range, with an average predicted minimum (maximum) slightly below 0.5 (1.0) across most models. Model 7.5 (MERF with all considered covariates) resulted in the widest range with an average lowest (highest) predicted value of 0.38 (0.99). For data availability scenarios 1–4, Tobit performed the best in terms of MSE, MAE and R^2^. For data availability scenario 5, MERF yielded the best performance measures.

**Table 3:**
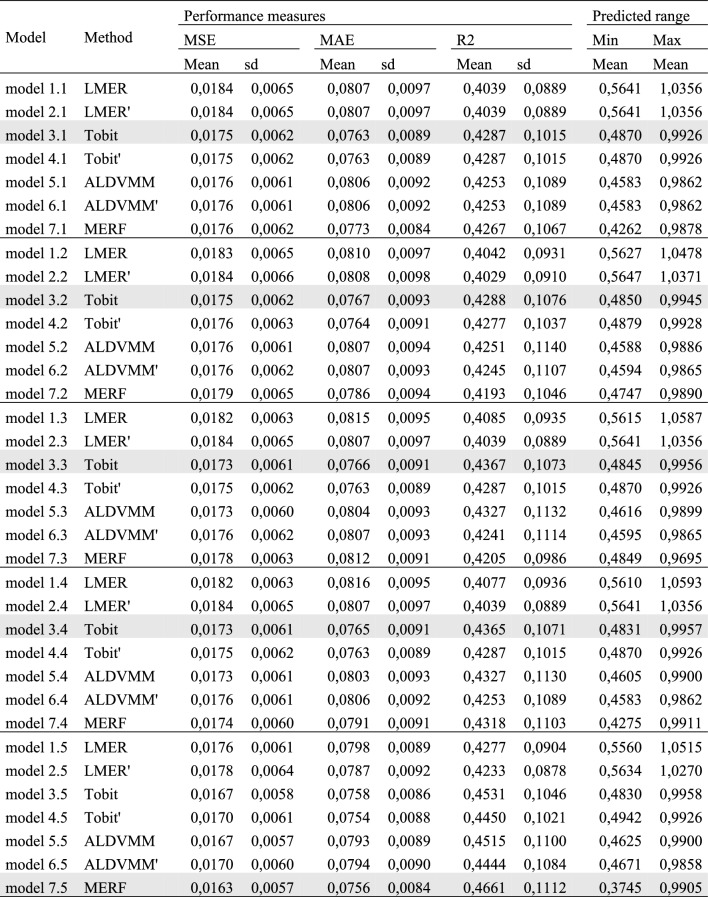
Performance measures from tenfold cross-validation

Best performing models for each set of covariates are marked in grey

*MAE* mean absolute error, *MSE* mean squared error, *LMER* linear mixed effects regression, *ALDVMM* adjusted limited dependent variable mixture model, *MERF* Mixed Effects Regression forest

The introduction of more covariates did not always improve predictions. As can be seen from our robustness checks in Online Appendix 2, performance measures improved when we did not include age and gender in the final models. Moreover, excluding years since initial diagnosis led to better results in terms of MSE, MAE and R^2^.

Based on this, we chose the following three final models:

- Final model 3.1, which is a mixed-effects Tobit regression with the general SIBDQ as the only covariate, was chosen for data availability scenarios 1 (only the general SIBDQ score is available) and 2 (general SIBDQ score, age and gender are available).
- Final model 3.3′, which is a mixed-effects Tobit regression with the covariates general SIBDQ score, BMI, smoking status and disease type, was chosen for data availability scenarios 3 (general SIBDQ score, BMI, smoking status and disease type) and 4 (BMI, smoking status, disease type and disease duration).
- Final model 7.5′, which is a MERF estimated with the covariates general SIBDQ score, BMI, smoking status, disease type and SIBDQ subscales, was chosen for data availability scenario 5.

**Fig. 2:**
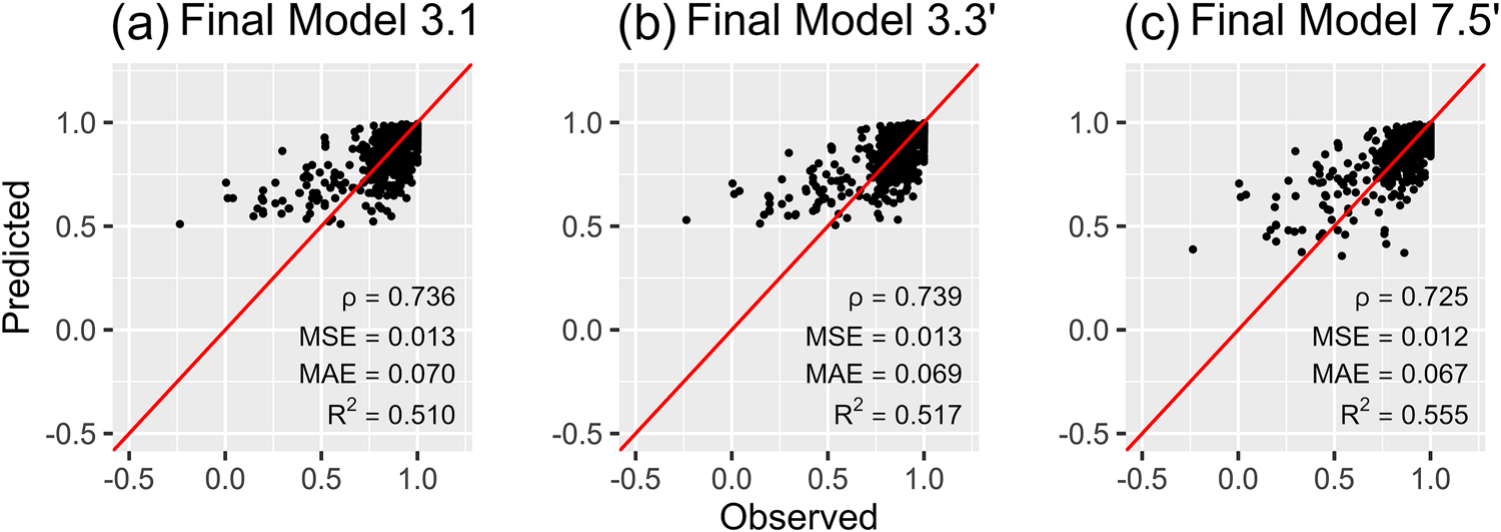
Scatterplots of the observed vs. predicted EQ-5D-5L utility index scores in the validation subset for the final models, **a** Mixed-effects Tobit regression with general SIBDQ score only, **b** Mixed-effects Tobit regression with general SIBDQ score, BMI, smoking status and disease type; **c** Mixed-effects regression forest with general SIBDQ score, BMI, smoking status, disease type and SIBDQ subscales; *ρ* Spearman correlation coefficient, *MAE* mean absolute error, *MSE* mean squared error

In Fig. 2, the EQ-5D-5L utility index scores predicted by our three final models are plotted against observed scores in the validation subset. While the Spearman correlation coefficient resulting from model 7.5′ was lower than those obtained from the other final models, MSE, MAE and R^2^ improved consistently with the number of covariates. Moreover, all performance measures were better than those obtained from tenfold cross-validation. Regression results and variable importance measures of the three final models can be found in Online Appendix 2.

The following link leads to the web application, which allows our final models to be applied for mapping: https://www.bwl.uni-hamburg.de/hcm/forschung/mapping.html.

## Discussion

The aim of our study was to develop a mapping algorithm that allows EQ-5D-5L estimates for health economic evaluation to be obtained from the disease-specific SIBDQ. To do so, we compared different direct mapping approaches, including LMER, mixed-effects Tobit regression, ALDVMM and MERF for different sets of covariates. We used data from 1055 adult IBD patients who had enrolled in a German randomised controlled trial.

Overall, there was a relatively strong correlation between SIBDQ and EQ-5D-5L, resulting in a Spearman correlation coefficient of 0.733 at baseline. We concluded that there was sufficient conceptual overlap between these two measures to develop a mapping algorithm.

Tobit regression performed best in four out of five considered data availability scenarios, while MERF had the best performance in the most complex data availability scenario. Specifying the regression models based on lasso often resulted in all covariates other than the general SIBDQ score being excluded from the model in most cases. Thus, the performance measures were mostly equal to the bivariate specification with the general SIBDQ score as the only explanatory variable, except when SIBDQ subscales were used for mapping.

The performance measures of the final models in the validation subset were better than those obtained from tenfold cross-validation, indicating that the model did not overfit the data.

The most important predictor for EQ-5D-5L in final model 7.5′ was the general SIBDQ score, followed by the SIBDQ Emotional and Social subscales. These reduced impurity by 15.5, 13.4 and 8.2, respectively. Our findings suggest that the mapping was not improved by the demographic variables age and gender, nor by information about time since initial diagnosis. Compared to other variables, the variable importance of disease subgroup, which reduced impurity by only 0.32, was low. We therefore concluded that providing a unique mapping algorithm for CD, UC and IC patients rather than estimating single mappings by subgroup would be sufficient.

Our results also indicate that using MERF to predict utility scores may improve outcomes compared to classical approaches when many covariates are available for mapping. Employing machine learning techniques for mapping is still uncommon [29]. However, some mapping studies have taken this path in recent years [41, 54, 55]. Because the core strength of machine learning is prediction, mapping studies appear to be a suitable application field for it. One could argue that the main focus of mapping algorithms should be prediction accuracy instead of interpretability, because no causal relationships are addressed. Moreover, practicability can be provided by embedding the model into a user-friendly environment, as we did with our web application. We would therefore welcome an increased use of machine learning methods in other mapping studies.

This study has several important limitations. First, we estimated the utility index score directly, because a crosswalk approach would have required a larger data set to ensure a sufficient share of the 3125 attainable health states of the EQ-5D-5L. Thus, conceptual dimensions of the SIBDQ are related to general population preferences based on the conceptual dimensions of the EQ-5D-5L. This may compromise the conceptual clarity of the relationship between the two measures [56].

Second, while the upper limit of OLS predictions exceeded the theoretical range, Tobit, ALDVMM and MERF tended to underpredict high health states. The lower part of the distribution, on the other hand, was systematically overpredicted by all models. The main explanation for these outcomes in the lower half of the distribution is that there were few observations with low health states in our data. However, the problem of over- and underprediction is common in mapping studies [24].

Third, the data set used to validate the final models was not independent of the data set used for model selection and estimation, because they were both subsamples of patients participating in the same RCT. Moreover, splitting the data set into a model and a validation subset came at the cost of a reduced sample size, which is why ISPOR guidelines currently do not recommend this approach [24]. However, in machine learning literature it is common practice to apply this form of sample splitting in order to obtain an honest evaluation of the final model performance [57]. In our case, we considered splitting the sample as appropriate since the size of the model subset was still comparable to or even larger than samples used in other mapping studies (e.g. [37]).

Fourth, the inclusion criteria of the clinical trial stipulated that patients must be adults and receiving or eligible for biologic therapy. This implies that our sample consisted exclusively of patients with a complicated disease course. Although the distribution of age, sex, SIBDQ and EQ-5D-5L was similar to that reported in studies with different inclusion criteria, the mapping may not be suitable for paediatric IBD patients or IBD patients receiving conventional medical treatment. Research exploring the performance of the mapping for different groups of patients is needed in order to decide whether our mapping algorithm can be recommended as a general approach to predict EQ-5D-5L values with the SIBDQ.

## Conclusion

We developed and provide an algorithm that maps SIBDQ values to EQ-5D-5L index scores for different sets of covariates. An online tool to use the mapping algorithm in research practice is available via the following link: https://www.bwl.uni-hamburg.de/hcm/forschung/mapping.html. Our study targets situations in which utility index scores are not directly available. However, measuring and reporting EQ-5D-5L index values directly should be the preferred option. Moreover, our results suggest that machine learning methods may be superior to traditional regression approaches for mapping applications of this nature.

### Supplementary Information

Below is the link to the electronic supplementary material.

Supplementary file1 (7Z 17 KB)

Supplementary file2 (DOCX 62 KB)
