# Supplementary material for: Mapping from SIBDQ to EQ-5D-5L for patients with inflammatory bowel disease
Source: Eur J Health Econ. 2023 Jun 27;25(3):539–48. doi: 10.1007/s10198-023-01603-9 (PMC10972987; doi:10.1007/s10198-023-01603-9)
Supplement: Supplementary file 2 — Supplementary file2 (DOCX 62 KB) [file 10198_2023_1603_MOESM2_ESM.docx]

## Appendix

**A.1 Link to the online depository of the web application**

The code of the R Shiny web application can be downloaded from the following depository:

<https://github.com/IsaMariaSteiner/SIBDQ-to-EQ-5D-5L-Mapping-App>

**A.2 Estimation of predicted utilities**

For LMER

Models were estimated with the lmer command of the lme4^[[1]](#footnote-1)^ package. Predicted values were obtained with the predict.lmer command.

For mixed-effects Tobit regression

Models were estimated with the censReg command that is included in the censReg package^[[2]](#footnote-2)^. The variable was inverted prior to estimation and the left limit was set to 0. Predicted values for the censored variable were obtained as follows:

$$E\left( Y^{inv} \right)=P\left( Y^{inv}>0 \right)\cdot E\left( Y^{inv} | Y^{inv}>0 \right)$$

with

$$P\left( Y^{inv}>0 \right)=\Phi\left( \frac{E({Y^{inv}}^{*})}{\sigma_{\nu}} \right)$$

and

$$E\left( Y^{inv} | Y^{inv}>0 \right)= E({Y^{inv}}^{*})+\sigma\cdot\lambda\left( \frac{E({Y^{inv}}^{*})}{\sigma_{\nu}} \right)$$

$E({Y^{inv}}^{*})$ corresponds to the predicted values for the latent (inverted) variable. $\sigma_{\nu}$ is the scale parameter of the tobit regression that was obtained from the model object. $\Phi\left( \cdot\right)$ is the cumulative distribution function of the normal distribution and $\lambda\left( \cdot\right)$ is the inverse Mills ratio, i.e. the probability density function divided by the cumulative distribution function of the normal distribution $\lambda\left( x \right)= \left( \frac{\phi(x)}{\Phi\left( x \right)} \right)$. The utility predictions were finally obtained by converting the variable to its original scale:

$\hat{Y}=E\left( Y \right)=\left( E\left( Y^{inv} \right)-1 \right)\cdot(-1)$

For ALDVMM

Models were estimated with the aldvmm command from the aldvmm package in R^[[3]](#footnote-3)^. The models were estimated with one component. The lower and upper limits were set to -0.661 and 0.974, as they represent the lowest and second highest value in the German value set respectively. Predictions were obtained with the predict.aldvmm command.

For MERF

MERF were defined according to Hajjem et al. (2014)^[[4]](#footnote-4)^ and Krennmair & Schmid (2022)^[[5]](#footnote-5)^:

$$y_{i}=f\left( X_{i} \right)+Z_{i}b_{i}+\varepsilon_{i},$$

$$b_{i}\sim N\left( 0,D \right), \varepsilon_{it}\sim N\left( 0,R_{i} \right), i=1,\ldots n,$$

Where $y_{i}$ is the $n_{i}\times1$ vector of responses for the $n_{i}$ observations of cluster (i.e. patient) $i$.

$X_{i}$ is the $n_{i}\times p$ matrix of fixed effects covariates. $Z_{i}$ is the $n_{i}\times q$ matrix of random effects covariates (in our case, however, we only estimated a random intercept).

$b_{i}$is the $q\times1$ vector of random effects for cluster $i$. $\varepsilon_{i}$ is the $n_{i}\times1$ vector of errors. $D$ is the covariance matrix of $b_{i}$ and $R_{i}$ is the covariance matrix of $\varepsilon_{i}$.

The function ${f(X_{i})}$is estimated with a regression forest. Regression forests are an ensemble method for multiple regression trees, as proposed by Breiman (2001). A regression tree is created by partitioning the data based on the values of the covariates $X_{i}$ in mutually exclusive regions. Within each tree, local predictions of the response variable are made based on the observations contained in the region. The regression forest then calculates the predictions as a weighted average over all tree predictions.

We used the R package SAEforest (Krennmair, 2022)^[[6]](#footnote-6)^ to estimate MERFS, which implements the Random forest with the R package *ranger* (Wright & Ziegler, 2017). We grew 1000 regression trees on bootstrap samples and used the variance of the predicted variable as splitting criterion. The hyperparameters ‘mtry’ and ‘min.node.size’ were tuned by applying a random grid search with 20% of all possible combinations. Predictions were obtained with the predict command provided in SAEforest.

**A.3 Definition of performance measures**

The Mean squared error (MSE), mean absolute error (MAE) and R^2^ were defined as follows:

$$MSE=\frac{\sum_{i=1}^{N^{te}} {(Y_{i}-\hat{Y}_{i})}^{2}}{N^{te}}$$

$$MAE=\frac{\sum_{i=1}^{N^{te}} |Y_{i}-\hat{Y}_{i}|}{N^{te}}$$

$$R^{2}=1-\frac{\mathrm{MSE}}{\sigma_{Y}^{2}}$$

With $N^{te}$ number of observations $i$ in the test set, $Y_{i}$dependent variable, $\hat{Y}_{i}$ predicted values of the dependent variable and $\sigma_{Y}^{2}$ variance of the dependent variable.

The measures can be interpreted as follows:

| Performance measure | Theoretical range | Interpretation |
| --- | --- | --- |
| MSE | $[ 0,+\infty]$ | lower = better |
| MAE | $[ 0,+\infty]$ | lower = better |
| R^2^ | $[ -\infty, 1 ]$ | higher = better;  negative values indicate that predictions are worse than the mean |

**A.4 Robustness checks:
Comparison of 10-fold cross-validated performance measures for Models 3.3 and 7.5**

|  | Included variables |  | Performance measures | | | | | | | |  | Predicted range | | |
| --- | --- | --- | --- | --- | --- | --- | --- | --- | --- | --- | --- | --- | --- | --- |
|  |  |  | MSE | |  | MAE | |  | R^2^ | |  | Min | Max | |
|  |  |  | Mean | sd |  | Mean | sd |  | Mean | sd |  | Mean | Mean | |
| Model 3.3 (Tobit) | |  |  |  |  |  |  |  |  |  |  |  |  | |
| original model | SIBDQ overall score, age, gender, BMI, smoking status |  | 0,0173 | 0,0061 |  | 0,0766 | 0,0091 |  | 0,4367 | 0,1073 |  | 0,4845 | 0,9956 | |
| without age and gender | SIBDQ overall score, BMI, smoking status |  | 0,0172 | 0,0061 |  | 0,0763 | 0,0088 |  | 0,4378 | 0,1032 |  | 0,4840 | 0,9951 | |
| Model 7.5 (MERF) | |  |  |  |  |  |  |  |  |  |  |  |  | |
| original model | SIBDQ overall score, age, gender, BMI, smoking status, disease type, years since initial diagnosis, SIBDQ subscales |  | 0,0163 | 0,0057 |  | 0,0756 | 0,0084 |  | 0,4661 | 0,1112 |  | 0,3745 | 0,9905 | |
| without age and gender | SIBDQ overall score, BMI, smoking status, disease type, years since initial diagnosis, SIBDQ subscales |  | 0,0163 | 0,0057 |  | 0,0752 | 0,0084 |  | 0,4661 | 0,1080 |  | 0,3768 | 0,9938 | |
| without age, gender, IBD type and disease duration | SIBDQ overall score, BMI, smoking status, IBD type, SIBDQ subscales |  | 0,0162 | 0,0058 |  | 0,0749 | 0,0088 |  | 0,4702 | 0,1112 |  | 0,3734 | 0,9904 | |
| Best performing models for each data availability scenario are marked in grey. | | | | | | | | | | | | | |  |

**A.5 Performance measures of the final models in the validation sample**

| Model | MSE | MAE | R^2^ | Min | Max |
| --- | --- | --- | --- | --- | --- |
| Final Model 7.1‘ | 0.0127 | 0.0697 | 0.5102 | 0.5103 | 0.9926 |
| Final Model 7.3‘ | 0.0126 | 0.0692 | 0.5171 | 0.5048 | 0.9964 |
| Final Model 7.5‘ | 0.0116 | 0.0672 | 0.5548 | 0.3565 | 0.9897 |

**A.6 Variable importance measures of final model 7.5’**

| Variable | Variable importance (mean decrease in impurity) |
| --- | --- |
| SIBDQ overall score | 15.4983 |
| BMI | 3.9094 |
| Smoker | 0.5342 |
| CD (ref: UC or IC) | 0.3236 |
| SIBDQ Subscale Bowel | 5.3227 |
| SIBDQ Subscale Emotional | 13.3719 |
| SIBDQ Subscale Social | 8.2066 |

**A.7 Regression coefficients and standard errors of final models 3.1 and 3.3’**

|  | Model 3.1 | Model 3.3‘ |
| --- | --- | --- |
| Intercept | 0.7270  (0.0127) | 0.6119  (0.0259) |
| SIBDQ overall score | -0.1249  (0.0024) | -0.1236  (0.0025) |
| BMI |  | 0.0036  (0.0007) |
| Smoker |  | 0.0101  (0.0096) |
| CD (ref: UC or IC) |  | 0.0232  (0.0099) |
| $log\sigma_{\mu}$ | -2.2175  (0.0240) | -2.2379  (0.0250) |
| $log\sigma_{\nu}$ | -2.0772  (0.0095) | -2.0796  (0.0099) |
| *Note: The coefficients and standard errors correspond to the inverted variable. Predicted values need to be re-inverted in the following way:* $(predicted value-1)\cdot(-1)$*;* $\sigma_{\mu}$ *is the standard deviation of the individual specific effects;* $\sigma_{\nu}$ *is the standard variation of the remaining disturbance.* | | |

**A.8 Completed Checklist of Items to Include When Reporting a Mapping Study**

| Section/topic | Item no. | Recommendation | Reported on page number |
| --- | --- | --- | --- |
| Title and abstract  Title  Abstract | 1  2 | Identify the report as a study mapping between outcome measures. State the source measure(s) and generic, preference-based target measure(s) used in the study.  Provide a structured abstract including, as applicable: objectives; methods, including data sources and their key characteristics, outcome measures used and estima­tion and validation strategies; results, including indica­tors of model performance; conclusions; and implica­tions of key findings. | 1  1 |
| Introduction  Study rationale  Study objective | 3  4 | Describe the rationale for the mapping study in the context of the broader evidence base.  Specify the research question with reference to the source and target measures used and the disease or population context of the study. | 1-2  2 |
| Methods  Estimation sample    External validation sample  Source and target measures  Exploratory data analysis  Missing data  Modelling approaches  Estimation of predicted scores   or utilities  Validation methods  Measures of model   per­formance | 5  6  7  8  9  10  11  12  13 | Describe how the estimation sample was identified, why it was selected, the methods of recruitment and data collection, and its location(s) or setting(s).  If an external validation sample was used, the ration­ale for selection, the methods of recruitment and data collection, and its location(s) or setting(s) should be described.  Describe the source and target measures and the methods by which they were applied in the mapping study.  Describe the methods used to assess the degree of conceptual overlap between the source and target measures.  State how much data were missing and how missing data were managed in the sample(s) used for the analyses.  Describe and justify the statistical model(s) used to develop the mapping algorithm.  Describe how predicted scores or utilities are estimated for each model specification.  Describe and justify the methods used to validate the mapping algorithm.  State and justify the measure(s) of model performance that determine the choice of the preferred model(s) and describe how these measures were estimated and applied. | 2  NA  2-3  5  2  3  3, appendix A.2  3-4  3,  appendix A.3 |
| Results  Final sample size(s)  Descriptive information    Model selection  Model coefficients  Uncertainty  Model performance and face   validity | 14  15  16  17  18  19 | State the size of the estimation sample and any validation sample(s) used in the analyses (including both number of individuals and number of observations).  Describe the characteristics of individuals in the sample(s) (or refer back to previous publications giving such information). Provide summary scores for source and target measures, and summarize results of analyses used to assess overlap between the source and target measures.  State which model(s) is(are) preferred and justify why this(these) model(s) was(were) chosen.  Provide all model coefficients and standard errors for the selected model(s). Provide clear guidance on how a user can calculate utility scores based on the outputs of the selected model(s).  Report information that enables users to estimate standard errors around mean utility predictions and individual-level variability.  Present results of model performance, such as measures of prediction accuracy and fit statistics for the selected model(s) in a table or in the text.  Provide an assessment of face validity of the selected model(s). | 2  4-5  4-5  Variable importance measures of model 7.5’ are provided in appendix A.6, regression coefficients and standard errors of models 3.1 and 3.3’ are provided in appendix A.7.  The models are provided together with the web application in appendix A.1  appendix A.1, web application  7,  appendix A.5 |
| Discussion  Comparisons with previous   studies  Study limitations  Scope of applications | 20  21  22 | Report details of previously published studies developing mapping algorithms between the same source and target measures and describe differences between the algorithms, in terms of model performance, predictions and coefficients, if applicable.  Outline the potential limitations of the mapping algorithm.  Outline the clinical and research settings in which the mapping algorithm could be used. | NA  7  7-8 |
| Other  Additional information | 23 | Describe the source(s) of funding and non-monetary support for the study, and the role of the funder(s) in its design, conduct and report. Report any conflicts of interest surrounding the roles of authors and funders. | 8 |

1. D. Bates, M. Maechler, B. Bolker, S. Walker. Fitting Linear Mixed-Effects Models using lme4. *Journal of
    Statistical Software. 67*(1), 1–48 (2015). [↑](#footnote-ref-1)
2. A. Henningsen. *CensReg: Censored Regression (Tobit) Models.* R package version 0.5-36 (2022).

   https://cran.r-project.org/web/packages/censReg/index.html. [↑](#footnote-ref-2)
3. M. Pletscher. *aldvmm: Adjusted Limited Dependent Variable Mixture Models*, R

   package version 0.8.4. (2021). <https://CRAN.R-project.org/package=aldvmm>. [↑](#footnote-ref-3)
4. A. Hajjem, , F. Bellavance & D. Larocque. Mixed-effects random forest for clustered data. *Journal of*

   *Statistical Computation and Simulation*, *84*(6), 1313-1328 (2014). [↑](#footnote-ref-4)
5. Krennmair, P., Schmid, T.: Flexible domain prediction using mixed effects random forests.

   Journal of the Royal Statistical Society Series C: Applied Statistics, 71(5), 1865–1894 (2022). [↑](#footnote-ref-5)
6. P. Krenmair. *SAEforest:* *Mixed Effect Random Forest for Small Area Estimation.* R

   package version 1.0.0. (2022). https://cran.r-project.org/web/packages/SAEforest/index.html. [↑](#footnote-ref-6)
